# Supplementary material for: Circulating miRNAs in Small Extracellular Vesicles Secreted by a Human Melanoma Xenograft in Mouse Brains
Source: Cancers (Basel). 2020 Jun 19;12(6):1635. doi: 10.3390/cancers12061635 (PMC7352810; doi:10.3390/cancers12061635)
Supplement: Supplementary file 1 [file cancers-12-01635-s001.zip › Supplementary figures.pdf]

cancers

MDPI

Article

Circulating miRNAs in Small Extracellular Vesicles Secreted by a Human Melanoma Xenograft in Mouse Brains

Loredana Guglielmi, Marta Nardella, Carla Musa, Ingrid Cifola, Manuela Porru, Beatrice Cardinali, Ilaria Iannetti, Chiara Di Pietro, Giulia Bolasco, Valentina Palmieri, Laura Vilardo, Nicolò Panini, Fabrizio Bonaventura, Massimiliano Papi, Ferdinando Scavizzi, Marcello Raspa, Carlo Leonetti, Germana Falcone, Armando Felsani and Igea D'Agnano

Supplementary Materials

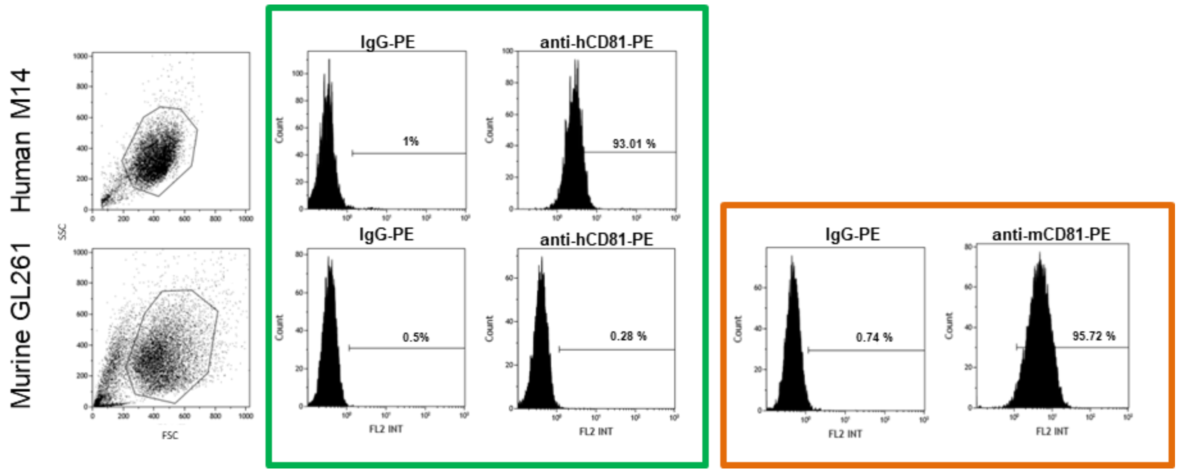

Figure S1. FACS analysis of the anti-hCD81-PE antibody on human and murine cells. We used the M14 human melanoma cell line as human model and the GL261 murine glioma cell line as mouse model. The green box encloses the histograms related to the test of the anti-human CD81 on the human M14 and murine GL261 cells specifically evidencing CD81 on human cells. The orange box encloses the histograms related to the test of an anti-mouse CD81 on the murine GL261 cells to evidence CD81 on murine cells as control. Corresponding IgG conjugated with phycoerythrin (PE) were used as control isotype in the negative samples. Debris and aggregates were excluded from the analysis by a gating process as shown on the left panels in the cytograms forward scatter (FSC)/side scatter (SSC). Ten-thousand events were acquired in each sample by a Gallios 8 color, equipped with 3 lasers (488nm Blue, 561 Yellow-Green and 638nm Red), Beckman Coulter Life Science, Indianapolis USA. The percentage in each histogram represents the fraction of positive cells.

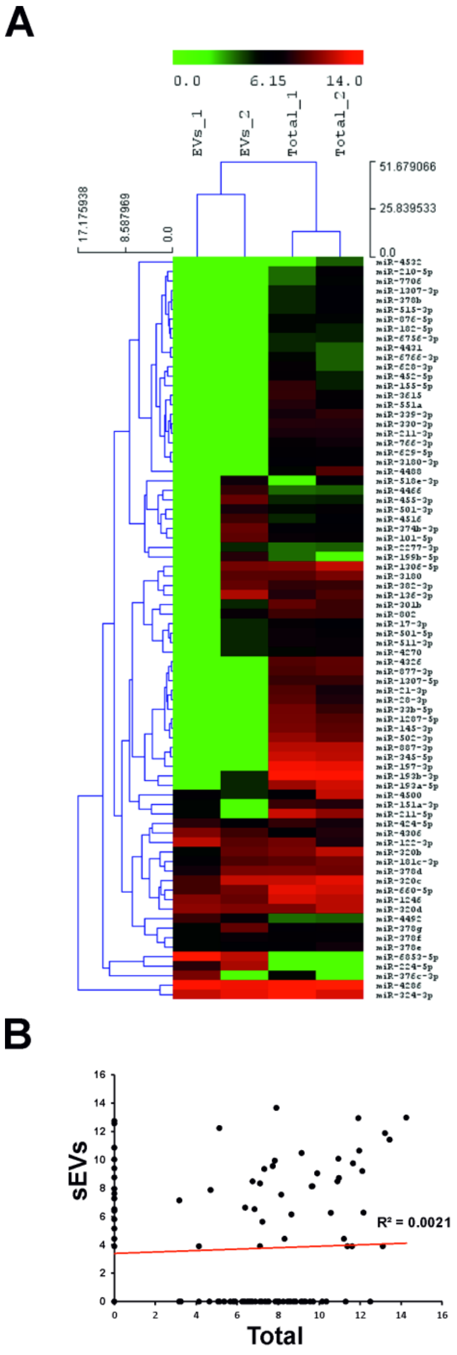

Figure S2. Hierarchical clustering analysis of the human-specific miRNAs released in the mice total plasma and enriched in the sEVs. (**A**) Clustering analysis was performed considering the data in the total plasma and sEV cargo at day 23 post-implantation. (**B**) Regression analysis between the two compartments, $R^2 = 0.0021$.

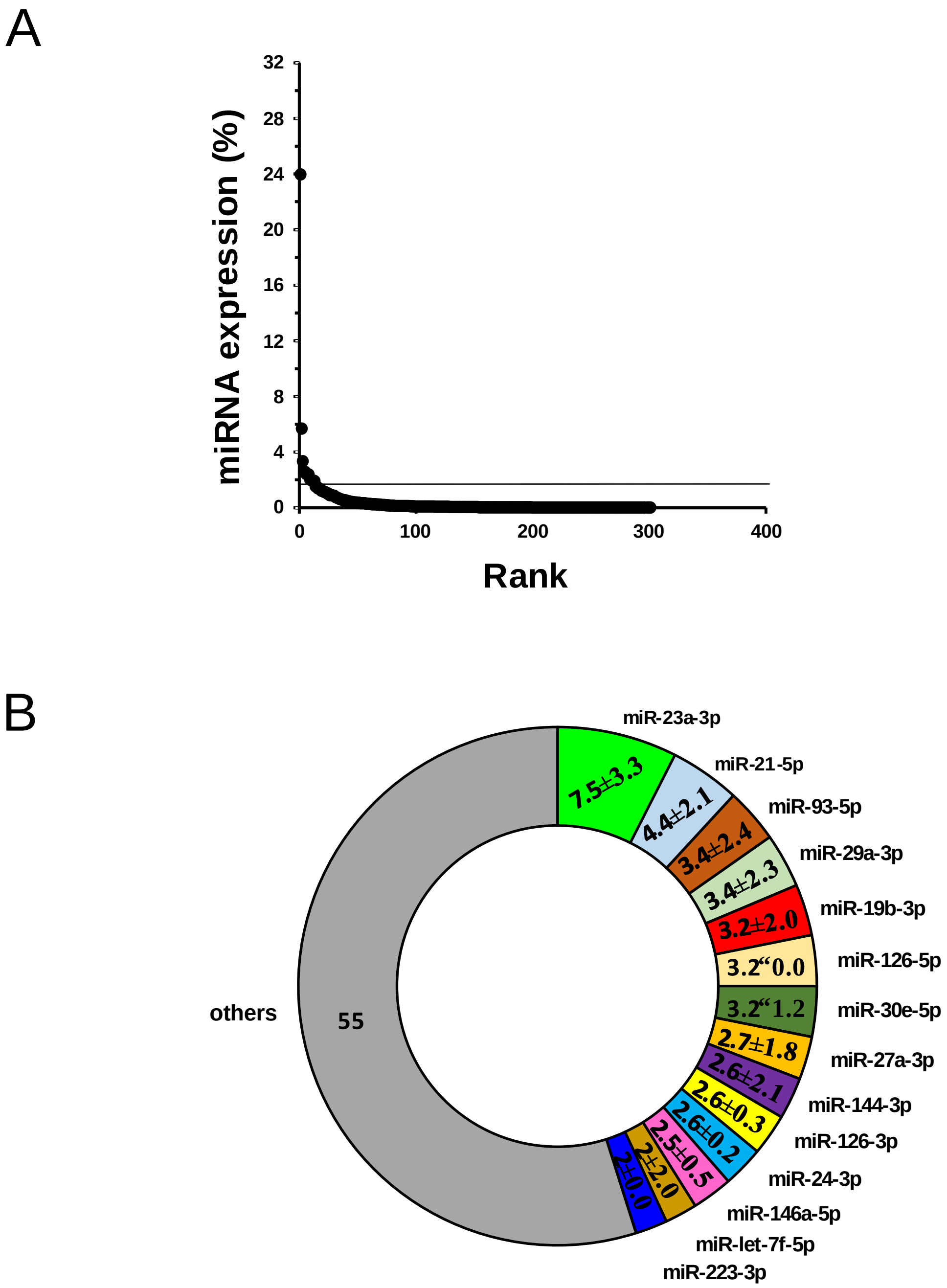

Figure S3. miRNA abundance analysis in cell cultures. (**A**) Rank abundance curve of the miRNAs expressed in the sEVs released in M14 cell culture medium. The threshold was set at the value of 2% miRNA expression (horizontal line). (**B**) The most abundant miRNAs are shown in the ring graph and the number reported in each slice of the ring represents the percentage of expression of the indicated miRNA.

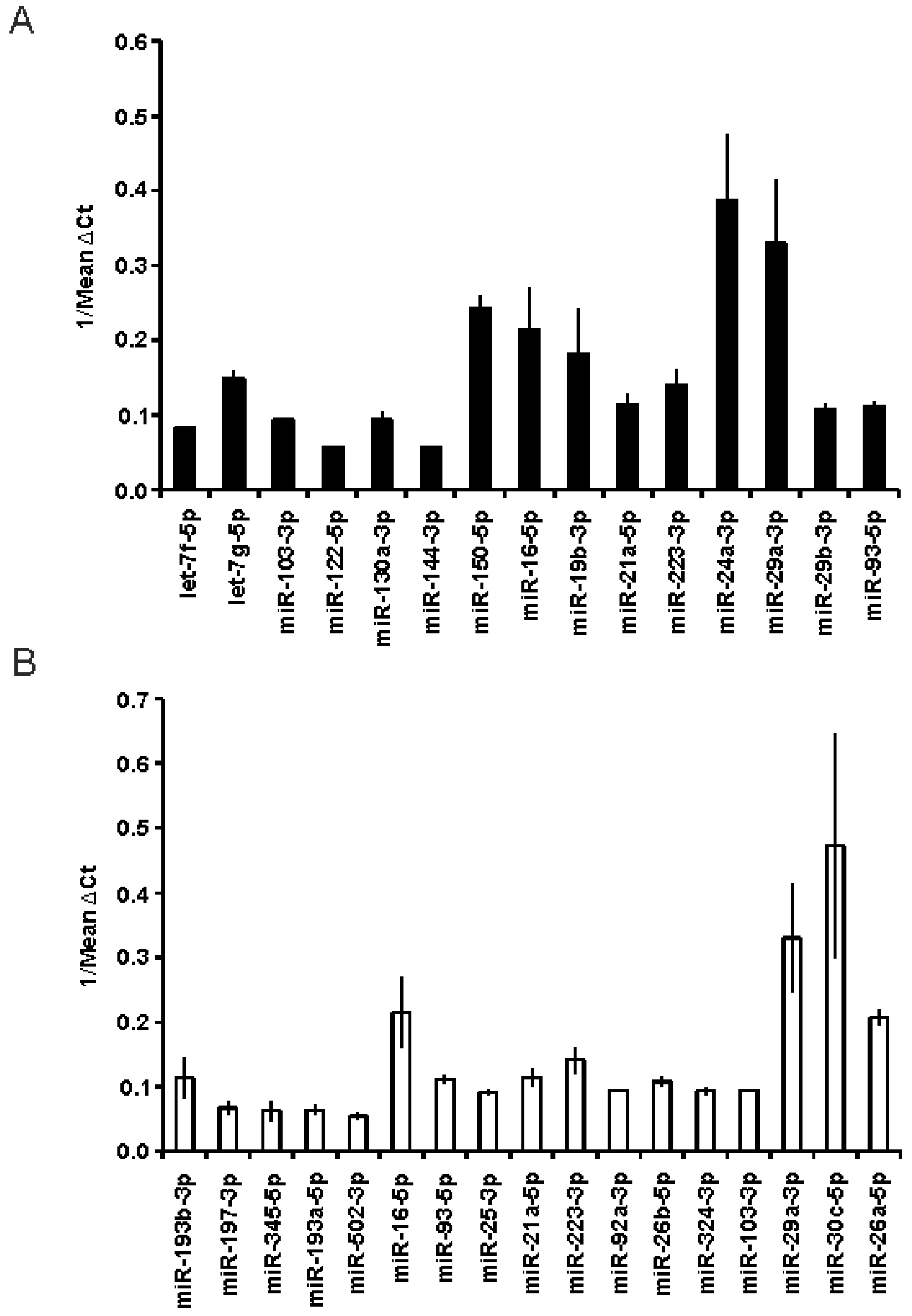

Figure S4. miRNA expression analysis by Taqman Array MicroRNA Cards in M14 melanoma tumor xenografts from mice brain. (**A**) Normalized expression levels of miRNAs found in sEV of our experimental model and expressed in M14 melanoma tumor xenograft. (**B**) Normalized expression levels of miRNAs found in total plasma of our experimental model and expressed in M14 melanoma tumor xenografts. Mean delta Ct (ΔCt) values have been calculated on two pool replicates, normalizing each miRNA on the average of two housekeeping genes. The higher the 1/Mean ΔCt value, the higher the miRNA expression. Bars represent standard error in each histogram.
